# Transcriptomic Insights into the Dual-Modulatory Role of EGCG in Alleviating Glyphosate-Induced Oxidative Stress in *Cucumis melo*

**DOI:** 10.3390/ijms26209887

**Published:** 2025-10-11

**Authors:** Qiuying Lu, Dongmiao Zhai, Yaxian Wu, Yihu Mao, Golam Jalal Ahammed, Xinzhong Zhang, Jingbo Yu, Xin Li

**Affiliations:** 1 College of Horticulture and Landscape Architecture, Tianjin Agricultural University, Tianjin 300384, China; 2 Key Laboratory of Tea Quality and Safety Control, Ministry of Agriculture and Rural Affairs, Tea Research Institute, Chinese Academy of Agricultural Sciences, Hangzhou 310008, Chinazxz.1982@163.com (X.Z.); 3 College of Horticulture and Plant Protection, Henan University of Science and Technology, Luoyang 471023, China; ahammed@haust.edu.cn; 4 College of Horticulture, Nanjing Agricultural University, Nanjing 210095, China

**Keywords:** EGCG, glyphosate, *Cucumis melo*, detoxification, flavonoid biosynthesis, RNA-Seq, antioxidant defense

## Abstract

Glyphosate is one of the most widely used herbicides in agricultural, horticultural, and urban environments. However, its residue accumulation and oxidative damage pose serious threats to crop health and food safety. In this study, we evaluated the potential of epigallocatechin gallate, a natural polyphenol derived from tea, to alleviate glyphosate-induced stress in melon (*Cucumis melo* L.). LC-MS/MS analysis revealed that EGCG significantly reduced glyphosate residues in plant tissues. Transcriptome analysis indicated that glyphosate induced extensive transcriptional reprogramming, activating genes involved in detoxification and antioxidant defense. Co-treatment with glyphosate and EGCG partially mitigated this stress response and redirected gene expression toward secondary metabolic pathways, particularly flavonoid and phenylalanine biosynthesis. Under herbicide stress, EGCG restored the transcription of key flavonoid biosynthetic genes, including *PAL*, *C4H*, *CHI*, and *OMT*. Meanwhile, EGCG also modulated the expression of *APX*, *SOD*, and *GST*, suggesting a selective effect on antioxidant systems. Co-expression network analysis identified key hub genes associated with oxidative stress and flavonoid metabolism. These findings demonstrate the dual regulatory role of EGCG in suppressing acute oxidative stress while enhancing metabolic adaptability, highlighting its potential as a natural additive for reducing herbicide residues in fruit crops.

## 1. Introduction

Melon (*Cucumis melo* L.) is a globally significant horticultural crop, highly valued for its refreshing taste, rich nutritional profile, and economic importance. It is particularly popular in summer due to its hydrating and cooling properties, contributing to its widespread cultivation and consumption worldwide [1,2]. In melon-growing regions, however, the presence of vigorous weed growth poses persistent agronomic challenges [3]. Weeds not only compete with crops for essential resources such as water, nutrients, and light but also serve as reservoirs for various insect pests and pathogenic microorganisms. These factors severely compromise crop health, reduce fruit yield and quality, and increase the difficulty of field management [4].

To mitigate weed pressure and reduce manual labor input, farmers often resort to the application of chemical herbicides for efficient weed control. Among these, glyphosate [N-(phosphonomethyl)glycine] is one of the most widely used non-selective, systemic herbicides in modern agriculture [5,6]. Its primary mode of action involves the inhibition of 5-enolpyruvylshikimate-3-phosphate synthase (EPSPS), a key enzyme in the shikimate pathway, ultimately disrupting the biosynthesis of aromatic amino acids such as phenylalanine, tyrosine, and tryptophan, which are essential for plant growth and development [7,8]. Despite its herbicidal efficacy, glyphosate and its major degradation product, aminomethylphosphonic acid (AMPA), are known to accumulate in non-target plant tissues through drift, leaching, or surface runoff [9,10]. This unintended exposure can cause a broad spectrum of adverse effects on crop physiology, including photosynthetic inhibition, disruption of carbon and nitrogen metabolism, impairment of mineral uptake, and induction of oxidative stress [11,12]. These stress symptoms are often associated with excessive reactive oxygen species (ROS) generation, mitochondrial dysfunction, and the suppression of critical metabolic and signaling pathways, ultimately threatening fruit quality and food safety [13,14].

In recent years, natural bioactive compounds have emerged as promising candidates for mitigating the negative effects of agrochemicals on crops [15,16]. EGCG, the most abundant catechin in green tea, has garnered significant attention due to its potent antioxidant activity and ability to modulate diverse physiological and biochemical processes [17,18]. Epigallocatechin gallate (EGCG) has been shown to scavenge ROS directly, enhance the activities of antioxidant enzymes such as superoxide dismutase (SOD), catalase (CAT), peroxidase (POD), ascorbate peroxidase (APX) and glutathione S-transferase (GST), and regulate redox-related transcription factors, including members of the WRKY and MYB families [19,20,21]. In tomato (*Solanum lycopersicum* L.), exogenous application of EGCG alleviated cadmium-induced stress by enhancing the activities of antioxidant enzymes, including CAT, SOD, APX, and POD, with increases ranging from 18.06% to 55.54% [22]. Moreover, EGCG has been implicated in the modulation of secondary metabolic processes, notably the flavonoid biosynthesis pathway, which plays a central role in oxidative stress mitigation, hormone signaling transduction, and plant defense activation [23,24]. Although the protective roles of EGCG against various abiotic stresses such as salinity, heavy metals, and heat stress have been well documented, its potential function under herbicide-induced oxidative stress remains largely unexplored. In particular, little is known about whether and how EGCG regulates secondary metabolism and antioxidant defense pathways to mitigate glyphosate toxicity in fruit crops like melon. Addressing this gap will provide new insights into the dual role of EGCG in alleviating herbicide phytotoxicity and promoting sustainable crop protection.

To address this knowledge gap, the present study aimed to investigate the transcriptional responses of the flavonoid biosynthetic pathway in melon subjected to EGCG treatment, glyphosate exposure, and their combination. Using high-throughput RNA sequencing (RNA-seq), we systematically analyzed global gene expression changes and identified differentially expressed genes (DEGs) involved in antioxidant defense and flavonoid metabolism. Functional annotation and enrichment analyses based on Gene Ontology (GO) and the Kyoto Encyclopedia of Genes and Genomes (KEGG) databases were conducted to explore the molecular mechanisms and signaling networks underlying EGCG-mediated mitigation of glyphosate-induced stress. The findings from this study offer new insights into the potential application of natural polyphenols for enhancing herbicide tolerance.

## 2. Results

### 2.1. Overview of RNA-Seq Data Quality and Read Mapping Efficiency Under Different Treatments

To investigate the transcriptional regulatory effects of exogenous EGCG and Gly on gene expression in melon, transcriptome sequencing was performed using the Illumina NovaSeq 6000 platform. Detailed RNA-seq data statistics are summarized in Table 1. A total of 88.25 GB of raw sequencing data was generated from 12 libraries, with an average of 7.35 GB per sample. After stringent quality control, 86.26 GB of clean data was retained (7.19 GB on average per sample). The quality scores of the clean reads were high, with Q20 and Q30 values of 97.77% and 94.78%, respectively, indicating excellent data reliability. The average GC content across all samples was 45.6%, consistent with the GC characteristics of the melon genome. Clean reads were then aligned to the reference genome using HISAT2. The alignment results showed that over 95% of the reads were uniquely mapped to the genome. In total, 570,934,820 high-quality, uniquely aligned reads were obtained from the 12 samples, with an average mapping rate of 97.22%. Only 2.78% of the reads remained unmapped (approximately 1,363,870 reads per sample), demonstrating high efficiency and accuracy of genome alignment.

### 2.2. Transcriptomic Differentiation and EGCG-Mediated Modulation of Glyphosate Accumulation in Melon Leaves Under Different Treatments

**Figure 1:**
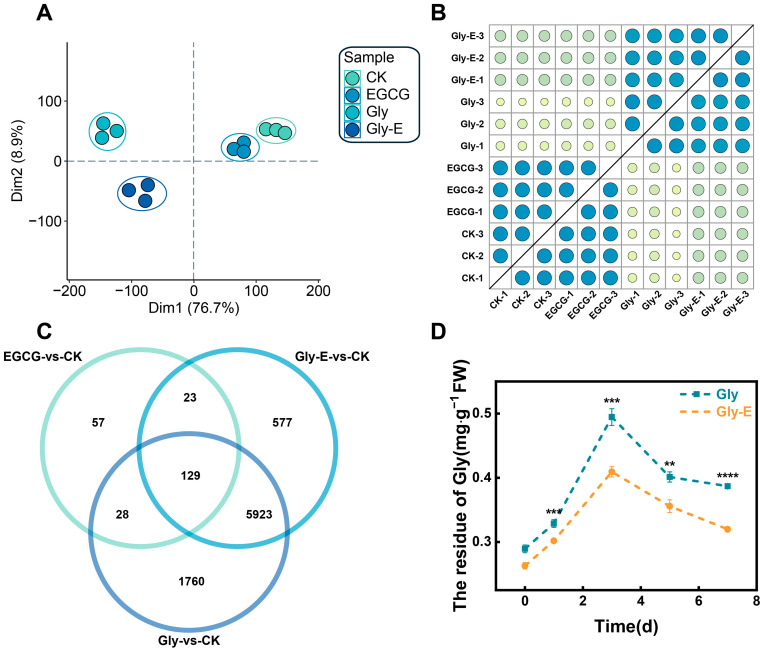
Transcriptome profiling and glyphosate residue dynamics in melon leaves under different treatments. (**A**) Principal component analysis (PCA) of all RNA-seq samples showing clear separation among treatment groups. PC1 and PC2 accounted for 76.7% and 8.9% of the total variance, respectively. (**B**) Pearson correlation heatmap illustrating high intra-group consistency across biological replicates (R^2^ > 0.80). (**C**) Venn diagram showing the overlap of DEGs among treatment comparisons (FDR < 0.05, |log_2_FC| ≥ 1). A total of 129 DEGs were commonly regulated across all groups, with 57, 1760, and 577 specific to EGCG, Gly, and Gly-E treatments, respectively. (**D**) Dynamic changes in glyphosate residues (mg·g^−1^ FW) in melon leaves over a 7-day period in Gly- and Gly-E-treated plants. Residue levels were significantly lower in the Gly-E group compared to Gly at all measured time points. Values represent means ± SD (n = 3). Statistical significance was determined by one-way ANOVA followed by LSD test: ** *p* < 0.01; *** *p* < 0.001; **** *p* < 0.0001.

To evaluate transcriptional differences among treatment groups and assess the reliability of the RNA-seq data, principal component analysis (PCA) was performed. The first two principal components, PC1 (18.77%) and PC2 (16.75%), clearly separated the samples by treatment (Figure 1A). Biological replicates within each group clustered tightly, while distinct grouping patterns were observed among treatments—CK and EGCG samples clustered together, as did Gly and Gly-E samples. These patterns indicate both the effective response to Gly treatment and the biological impact of EGCG application.

In addition, sample correlation analysis confirmed high reproducibility across biological replicates, with all Pearson correlation coefficients (R^2^) exceeding 0.80 (Figure 1B). Venn diagram analysis identified 129 DEGs commonly responsive to all treatments (FDR < 0.05, |log_2_FC| ≥ 1), as well as treatment-specific DEGs: 57 in the EGCG group, 1760 in the Gly group, and 577 in the Gly-E group (Figure 1C).

**Figure 2:**
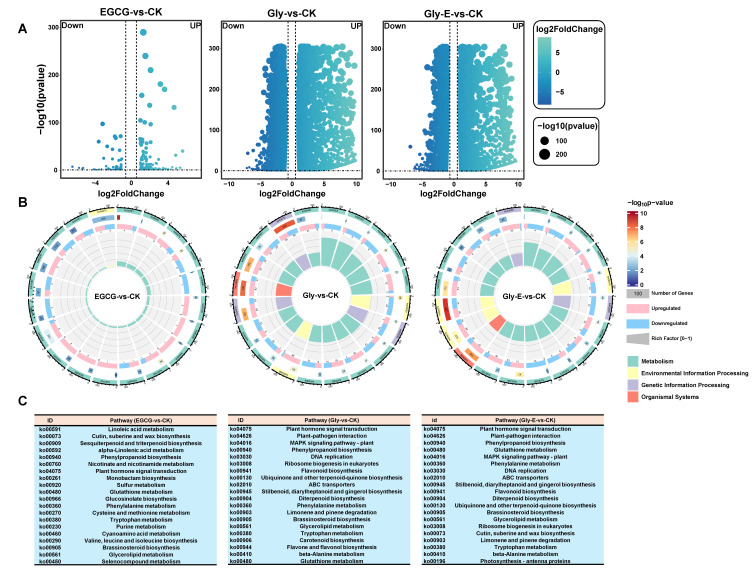
Differential gene expression and KEGG pathway enrichment under different treatments. (**A**) Volcano plots showing DEGs in three pairwise comparisons: EGCG vs. CK, Gly vs. CK, and Gly-E vs. CK. *X*-axis represents log_2_(fold change), and *Y*-axis shows −log_10_ (*p*-value). Point size indicates statistical significance, and color scale indicates fold change magnitude. (**B**) Circular diagrams illustrate KEGG pathway enrichment of DEGs in each treatment group. The outermost ring color codes the functional category of each pathway: green for metabolism, yellow for environmental information processing, purple for genetic information processing, and orange for organismal systems. The second ring represents the enrichment significance: dark red bars indicate higher enrichment (lower *p*-values), while dark blue bars indicate lower enrichment; the bar length corresponds to the number of DEGs in each pathway. The third ring shows the proportion of upregulated (pink) and downregulated (blue) genes within each pathway. The innermost ring repeats the functional classification of pathways, with bar lengths again representing the gene count. (**C**) Top 20 significantly enriched KEGG pathways (FDR < 0.05) identified in each treatment group. Pathways are ranked by adjusted *p*-value.

To further examine the regulatory effect of EGCG on glyphosate accumulation, glyphosate residue levels were monitored over a 7-day period in leaves from Gly- and Gly-E-treated plants (Figure 1D). As shown in Figure 2B, glyphosate residues increased rapidly within the first 3 days in both groups, peaking on day 3. However, throughout the entire course, residue levels in the Gly-E group remained significantly lower than those in the Gly group. A significant reduction was observed as early as day 0 (*p* < 0.001), and the difference persisted at day 3 (*p* < 0.001), day 5 (*p* < 0.01), and day 7 (*p* < 0.0001). These results suggest that exogenous EGCG application may facilitate glyphosate metabolism or reduce its accumulation in plant tissues.

### 2.3. Transcriptome-Wide Differential Gene Expression and KEGG Pathway Enrichment Under Different Treatments

Volcano plot analysis revealed the patterns of DEGs across treatment groups (Figure 2A). The CK and EGCG treatments resulted in 156 upregulated and 81 downregulated genes. Gly treatment induced a substantial transcriptional shift, with 4209 genes upregulated and 3631 genes downregulated. Under the Gly-E treatment, the overall magnitude of gene expression disturbance was reduced relative to Gly alone, yet a large number of DEGs were still detected, including 3678 upregulated and 2974 downregulated genes.

KEGG pathway enrichment analysis demonstrated treatment-specific regulatory patterns (Figure 2B). In the EGCG group, 19 of the top 20 significantly enriched pathways were metabolism-related, including phenylpropanoid biosynthesis, while only one pathway—plant hormone signal transduction (ko04075)—belonged to the environmental information processing category (Figure 2C). In contrast, the Gly treatment group displayed a more complex enrichment profile. Among the top 20 pathways, three were associated with environmental signaling (plant hormone signal transduction, ABC transporters [ko02010], and MAPK signaling [ko04016]), two were involved in genetic information processing (DNA replication [ko03030] and eukaryotic ribosome biogenesis), and one belonged to the organismal systems category (plant–pathogen interaction [ko04626]). The remaining 14 were metabolism-related pathways.

The KEGG category distribution in the Gly-E group was largely similar to that of the Gly group, though the specific types of enriched metabolic pathways differed. Notably, phenylpropanoid biosynthesis (ko00940), phenylalanine metabolism (ko00360), and flavonoid biosynthesis (ko00941) were significantly enriched across all three treatment groups. Importantly, both phenylpropanoid and phenylalanine metabolism pathways showed significantly higher enrichment in the Gly-E group than in the Gly group alone (*p* < 0.05), suggesting that EGCG may facilitate glyphosate detoxification by enhancing secondary metabolic pathways.

### 2.4. GO Functional Enrichment Reveals Treatment-Specific Biological Responses Under Different Treatments

**Figure 3:**
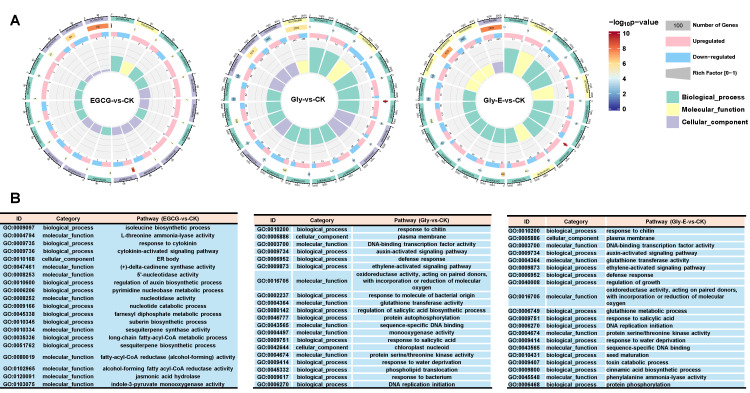
GO enrichment analysis of DEGs under different treatments. (**A**) Circular diagrams representing Gene Ontology (GO) enrichment results for DEGs in EGCG vs. CK, Gly vs. CK, and Gly-E vs. CK comparisons. GO terms are grouped into three categories: biological process (green), molecular function (purple), and cellular component (yellow). The outermost ring indicates the GO category. The second ring color scale reflects the significance level of enrichment (−log_10_ *p*-value). The third ring displays the proportion of upregulated (pink) and downregulated (blue) DEGs for each term. The innermost ring indicates the number of DEGs enriched per GO term. (**B**) Tables showing selected top-enriched GO terms for each treatment group. Each entry includes the GO ID, functional category, and pathway description.

GO enrichment analysis revealed treatment-specific regulatory mechanisms in gene function across groups (Figure 3A). In the EGCG-treated group (EGCG vs. CK), DEGs were significantly enriched in biological processes such as isoleucine biosynthetic process (GO:0009097), cytokinin signaling pathway (GO:0009735/0009736), and regulation of auxin biosynthesis (GO:0010600), suggesting that EGCG may influence physiological functions by modulating amino acid metabolism and hormone signaling. Enriched molecular functions included L-threonine ammonia-lyase activity (GO:0004794), terpene synthase activity (GO:0010334), and fatty acid reductase activity (GO:0080019), indicating enhanced secondary metabolite biosynthesis. At the cellular component level, DEGs were mainly enriched in endoplasmic reticulum body (GO:0010168), which may be related to the storage or transport of secondary metabolites (Figure 3B).

In the glyphosate-treated group (Gly vs. CK), enriched GO terms were predominantly related to stress responses, including defense response (GO:0006952), DNA replication initiation (GO:0006270), and response to molecules of bacterial origin (GO:0002237). Upregulated molecular functions included glutathione transferase activity (GO:0004364) and oxidoreductase activity (GO:0016705), reflecting a coordinated detoxification and oxidative stress response. Enriched cellular components included the plasma membrane (GO:0005886) and chloroplast nucleoid (GO:0042644), which may participate in signal transduction and photosynthetic damage repair.

In the combined treatment group (Gly-E vs. CK), GO enrichment retained key defense-related processes seen in the Gly group (e.g., GO:0006952, GO:0006270) and additionally revealed responses to water deprivation (GO:0009414) and phenylalanine ammonia-lyase activity (GO:0045548). Molecular function analysis further indicated an upregulation of genes involved in the phenylpropanoid metabolic process (GO:0045548), suggesting that EGCG may alleviate glyphosate-induced toxicity by activating secondary metabolic pathways such as phenylpropanoid and glutathione metabolism. All three groups shared enrichment in hormone-related processes, including cytokinin and auxin signaling, whereas phenylpropanoid-related terms were uniquely and strongly enriched in the Gly-E group, highlighting EGCG’s potential role in regulating environmental stress adaptation mechanisms.

### 2.5. Differential Regulation of Flavonoid Biosynthesis Genes Under Different Treatments

**Figure 4:**
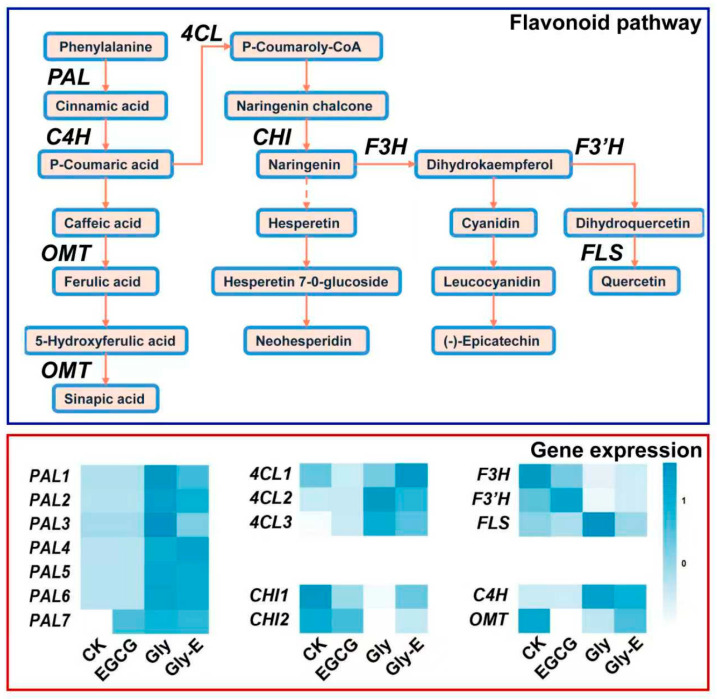
Expression profiles of key genes involved in the flavonoid biosynthesis pathway under different treatments. The blue box shows the flavonoid biosynthesis pathway, with orange solid arrows representing direct metabolic pathway flows, and dashed arrows indicating the presence of multiple steps in the pathway. The red box shows the gene expression heatmap, where the color gradient from blue to white indicates the relative transcript expression levels from high to low.

To clarify the regulatory effects of different treatments on the flavonoid biosynthesis pathway in melon, a pathway diagram was constructed based on transcriptome data. As shown in Figure 4, *Phenylalanine Ammonia-Lyase* (*PAL*) gene expression was significantly downregulated in both CK and EGCG treatments, indicating that the upstream phenylpropanoid pathway remained inactive under these conditions. In contrast, Gly treatment strongly upregulated *PAL* gene expression, highlighting its role as a potent inducer of this pathway. In the Gly-E treatment, *PAL* gene expression remained elevated but was consistently lower than in the Gly group, suggesting that EGCG partially counteracts the glyphosate-induced activation.

EGCG treatment suppressed *4-coumaroyl-CoA 1* (*4CL1*) gene expression while significantly enhancing the expression of *4CL2* and *4CL3*. Glyphosate strongly induced *4CL2* and *4CL3*, while *4CL1* remained at baseline levels. Under Gly-E treatment, *4CL1* expression was markedly upregulated, fully reversing its suppression under glyphosate alone. Meanwhile, the induction of *4CL2* and *4CL3* was reduced but remained higher than in the control, indicating differential modulation of *4CL* genes.

EGCG significantly downregulated *Chalcone Isomerase 1* (*CHI1*) gene expression and showed a slight inhibitory trend on *CHI2*. Glyphosate suppressed both *CHI* gene expression, while EGCG in the combined treatment alleviated this suppression and restored their expression to near-control levels.

The upstream gene expression of *Cinnamate-4-Hydroxylase* (*C4H*) was strongly induced by glyphosate, while this induction was partially attenuated in the Gly-E group. *Flavonol Synthase* (*FLS*) was repressed in the control group but significantly upregulated under glyphosate treatment. Notably, this glyphosate-induced activation was fully reversed by EGCG in the Gly-E group. The modifying gene *O-Methyltransferase* (*OMT*) was significantly downregulated under EGCG and further suppressed under glyphosate treatment, but its expression was reactivated in the Gly-E group, suggesting a compensatory regulatory effect.

*Flavanone 3-Hydroxylase* (*F3H*) and *Flavonoid 3′-Hydroxylase* (*F3′H*), which encode hydroxylases in the flavonoid pathway, were significantly downregulated in the Gly group and remained suppressed in the Gly-E group, indicating that EGCG failed to restore their expression. Interestingly, the expression pattern of *FLS* resembled that of *4CL*, showing opposite regulatory responses under glyphosate and combined treatments.

**Figure 5:**
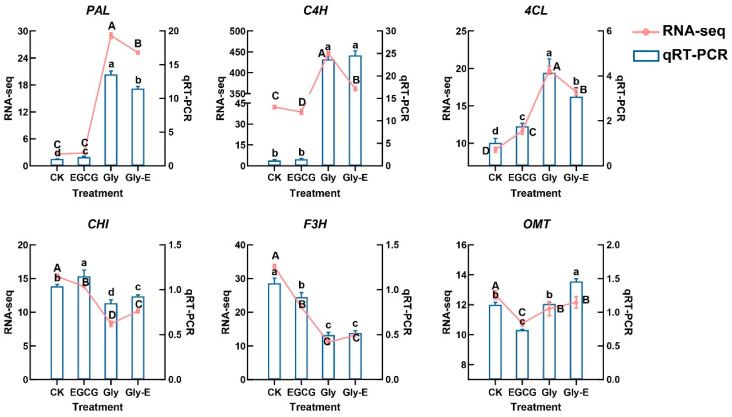
Validation of RNA-seq data by qRT-PCR for key genes in the flavonoid biosynthesis pathway under different treatments. Expression levels of six representative genes were analyzed using RNA-seq (red lines) and qRT-PCR (blue bars) in four different treatments. Left *Y*-axis represents RNA-seq data (FPKM), and right *Y*-axis corresponds to qRT-PCR results (relative expression). Data are presented as mean ± SD (n = 3). Different lowercase and uppercase letters indicate statistically significant differences (*p* ≤ 0.05) within qRT-PCR and RNA-seq results, respectively.

To validate the reliability of transcriptome sequencing data, six representative genes involved in the flavonoid biosynthesis pathway (*PAL*, *C4H*, *4CL*, *CHI*, *F3H*, and *OMT*) were selected for qRT-PCR analysis. The expression patterns obtained from qRT-PCR were highly consistent with the RNA-seq results, exhibiting similar trends in all treatment groups (Figure 5). This concordance confirms the accuracy and reproducibility of the RNA-seq data and supports the transcriptomic findings regarding the regulation of flavonoid-related genes under different treatments.

### 2.6. EGCG Selectively Modulates Antioxidant Gene Expression Triggered by Glyphosate Under Different Treatments

**Figure 6:**
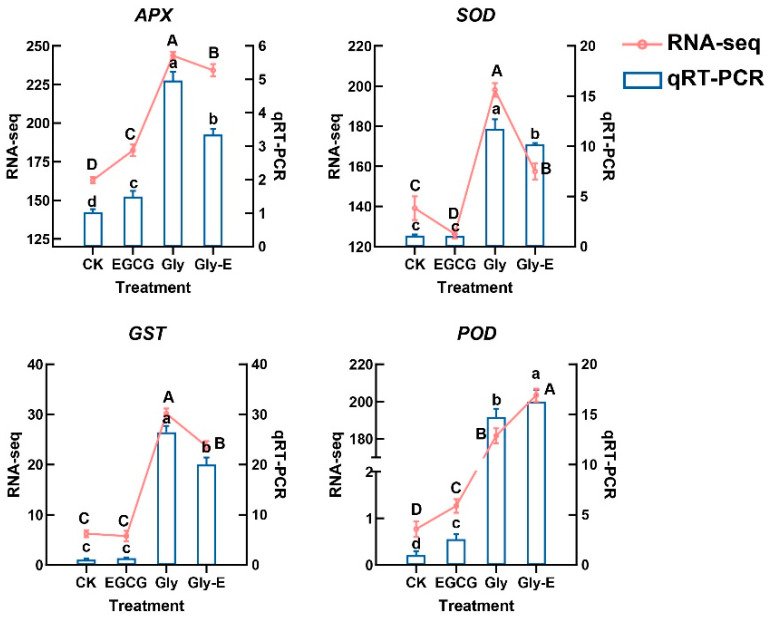
Validation of RNA-seq results by qRT-PCR for antioxidant-related genes under different treatments. Expression levels of four antioxidant-related genes were analyzed using RNA-seq (red lines) and qRT-PCR (blue bars) in four different treatments. Left *Y*-axis represents RNA-seq data (FPKM), and right *Y*-axis corresponds to qRT-PCR results (relative expression). Values are presented as mean ± SD (n = 3). Different lowercase and uppercase letters indicate statistically significant differences (*p* ≤ 0.05) within qRT-PCR and RNA-seq results, respectively.

APX, a key enzyme in the ascorbate-glutathione (AsA-GSH) cycle, plays a central role in H_2_O_2_ detoxification. Compared with CK treatment, *APX* gene expression was significantly elevated by 45.4% under EGCG treatment (*p* < 0.05). Gly treatment strongly induced *APX* gene expression to 4.8-fold higher than CK treatment (*p* < 0.05). However, in the Gly-E treatment, *APX* gene expression remained significantly higher than CK (*p* < 0.01) but was reduced by 32.6% compared to Gly (*p* < 0.05), suggesting that EGCG partially antagonized the glyphosate-induced upregulation of *APX* (Figure 6).

SOD, another core component of the antioxidant defense system, was slightly but significantly downregulated by 4.5% under EGCG treatment (*p* < 0.05), while Gly treatment dramatically upregulated *SOD* gene expression to 10.7-fold of CK (*p* < 0.05). Under Gly-E treatment, *SOD* gene expression remained significantly elevated compared to CK (*p* < 0.01) but was 13.3% lower than in the Gly group (*p* < 0.05), indicating a partial attenuation of glyphosate’s effect by EGCG.

GST, a key enzyme involved in detoxification under oxidative stress, was significantly upregulated by 28.2% following EGCG treatment (*p* < 0.05). Gly treatment induced a much stronger response, increasing *GST* gene expression to 21.8-fold of CK treatment (*p* < 0.01). Under Gly-E treatment, *GST* gene expression was 6.2% lower than in the Gly treatment (*p* < 0.05), suggesting that EGCG also antagonized glyphosate-induced *GST* activation.

POD, an important antioxidant enzyme, was significantly upregulated by 32.6% under EGCG treatment (*p* < 0.05), and further induced to 16.1-fold of CK under Gly treatment (*p* < 0.01). However, no significant difference in *POD* gene expression was observed between the Gly and Gly-E groups (*p* > 0.05), indicating that EGCG had no noticeable impact on the glyphosate-induced expression of *POD*.

Together, these results demonstrate that EGCG selectively modulates glyphosate-triggered antioxidant gene expression, exerting antagonistic effects on the *APX*, *SOD*, and *GST* pathways, but having limited influence on *POD*. This highlights a potential mechanism by which EGCG differentially regulates antioxidant defense genes in response to exogenous stress.

### 2.7. K-Means Clustering and Co-Expression Network Analysis of Antioxidant- and Flavonoid-Related Genes Under Different Treatments

**Figure 7:**
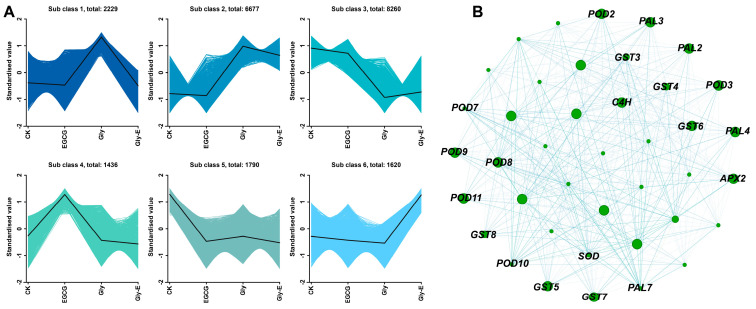
K-means clustering and co-expression network analysis of DEGs under different treatments. (**A**) K-means clustering of DEGs identified six distinct expression patterns (Subclasses 1–6) across treatment groups (CK, EGCG, Gly, Gly-E). Each subplot displays standardized expression values, with the black line representing the average trend per cluster. (**B**) Gene co-expression network constructed from DEGs with expression patterns similar to antioxidant and flavonoid biosynthesis genes. Node size reflects connectivity, and edges indicate co-expression relationships. Key genes related to antioxidant defense and secondary metabolism are labeled.

To further explore transcriptomic response patterns under different treatments, K-means clustering analysis was performed based on the expression profiles of DEGs. As shown in Figure 7A, the DEGs were divided into six distinct clusters (Subclasses 1–6), each displaying a characteristic expression trend across treatments. Among them, clusters showing upregulation in the Gly group and attenuation in the Gly-E group were of particular interest, as they were consistent with the expression trends observed for both antioxidant enzyme genes and flavonoid biosynthesis genes (Subclasses 1 and 2).

To identify key genes co-regulated with these functional groups, DEGs from clusters with expression trends similar to antioxidant and flavonoid genes were selected for co-expression network analysis. As shown in Figure 7B, the resulting gene co-expression network revealed strong interconnections among members of the *POD*, *GST*, *SOD*, *PAL*, *C4H*, and *APX* families. The size and connectivity of the nodes suggest potential hub genes that may play pivotal roles in the regulation of oxidative stress responses and secondary metabolism under glyphosate and EGCG treatments.

## 3. Discussion

For decades, pesticides have played an essential role in modern agricultural systems by effectively controlling pests, diseases, and weeds, thereby preventing crop losses and contributing significantly to global food security [25,26]. However, with the intensification of agricultural practices and the increasing demand for food products, farmers have become increasingly reliant on chemical pesticides. This overuse has raised serious environmental and food safety concerns, particularly the excessive accumulation of pesticide residues in crops, which poses growing risks to human health [27]. In our previous study, physiological and biochemical analyses demonstrated that exogenous application of EGCG significantly enhanced the degradation of glyphosate and carbendazim in melons. This effect was likely associated with the activation of antioxidant detoxification pathways mediated by GSH, suggesting a potential regulatory role for EGCG in pesticide metabolism [28]. To further investigate the molecular mechanisms underlying this phenomenon, we performed high-throughput transcriptome sequencing to comprehensively examine the regulatory effects of EGCG on gene expression in melon under glyphosate stress. The results revealed that flavonoid metabolism was markedly disrupted, indicating a substantial impact of glyphosate on plant metabolic networks. Moreover, the data supports the potential of EGCG as a natural bioactive compound capable of modulating endogenous detoxification pathways and reducing pesticide residues in crop tissues.

This study demonstrated that exogenous application of EGCG significantly accelerated the degradation of glyphosate in melon, reducing its residual concentration in leaf tissues and modulating associated detoxification metabolic pathways. During the 7-day monitoring period, glyphosate accumulation in the Gly-E combined treatment group was consistently and significantly lower than that in the glyphosate-only group at all points, indicating that EGCG has the capacity to promote glyphosate metabolism and elimination. This degradation-promoting effect is consistent with previous findings on exogenous additives regulating pesticide metabolism in tea [29] and tomato [30]. These additives are thought to enhance detoxification capacity by increasing antioxidant enzyme activity and upregulating detoxification-related gene expression. However, the current study suggests that the mechanism of EGCG may be more complex: in the Gly-E treatment, expression levels of classical detoxification genes such as APX and GST were lower than in the glyphosate-only group. This result indicates that EGCG may not alleviate toxicity solely through the activation of conventional detoxification pathways but may also coordinate alternative mechanisms to assist pesticide degradation in plants.

Plants commonly undergo widespread transcriptomic reprogramming in response to herbicide stress [31]. In this study, glyphosate exposure to melon led to the significant upregulation of over 7000 genes, activating a variety of pathways associated with redox homeostasis, detoxification metabolism, and signal transduction. This response was consistent with previously reported transcriptomic changes induced by herbicides in *Eleusine indica*, rapeseed (*Brassica napus* L.), and tea (*Camellia sinensis* L.) [18,32,33]. Co-application of EGCG substantially mitigated the acute transcriptional activation induced by glyphosate and shifted the expression profile toward secondary metabolism and adaptive regulation, with enrichment observed in pathways such as flavonoid biosynthesis, phenylpropanoid metabolism, and response to water deprivation. This transcriptional “buffering effect” has also been reported in other plant systems treated with exogenous melatonin [16], brassinosteroids [34], or salicylic acid [15]. GO enrichment analysis further supported this trend, revealing that the Gly group was primarily enriched in acute stress-related processes such as defense response (GO:0006952) and oxidoreductase activity (GO:0016705), while the Gly-E group showed additional enrichment in phenylpropanoid biosynthesis and water deprivation response pathways, which are associated with long-term metabolic remodeling. Furthermore, studies have shown that natural polyphenols can participate in transcriptional regulation by influencing MAPK cascades and plant hormone signaling pathways [35]. In our study, EGCG treatment significantly enriched GO terms associated with cytokinin and auxin signaling, further suggesting that EGCG may act as a regulatory molecule mediating transcriptional reprogramming. This shift from “acute defense” to “homeostatic regulation” and “metabolic adaptation” has also been documented in transcriptomic studies of herbicide and heavy metal stress responses in various plant species [36,37]. Importantly, these transcriptomic signatures can be further contextualized by recent insights into redox regulation. ROS and RNS are not only transient stress signals but also play pivotal roles in regulating chromatin dynamics through DNA methylation, histone modifications, and small RNA pathways, thereby stabilizing stress-responsive transcriptional states and contributing to stress memory. In particular, ROS such as H_2_O_2_ can activate MAPK cascades and coordinate crosstalk with phytohormone signaling [38], which closely parallels the enrichment of MAPK- and hormone-related GO terms observed under EGCG treatment. Thus, EGCG, by virtue of its strong redox-modulatory capacity, may act upstream of transcriptional reprogramming and reinforce adaptive homeostasis via conserved antioxidant mechanisms [38].

Flavonoids, as vital secondary metabolites, play crucial roles in antioxidant regulation, signal transduction, and environmental adaptation in plants [39,40]. Our study found that EGCG significantly regulated the expression of genes involved in the flavonoid biosynthesis pathway under glyphosate stress, indicating a reconstruction and activation of this metabolic route. Specifically, glyphosate treatment markedly induced the expression of upstream key enzymes such as *PAL*, *C4H*, and *FLS*, which may represent a compensatory response by the plant to oxidative stress through activation of defense-related signaling pathways. However, several downstream structural genes in the flavonoid biosynthesis pathway were generally repressed, suggesting that although glyphosate can initiate early signaling events, it may inhibit downstream metabolic flux due to its interference with aromatic amino acid biosynthesis, thereby disrupting the normal accumulation of flavonoids and other secondary metabolites.

In contrast, co-treatment with Gly-E significantly alleviated the repression of key structural genes such as *CHI* and *OMT*, while enhancing the enrichment of representative secondary metabolic pathways, including phenylpropanoid metabolism (ko00940) and flavonoid biosynthesis (ko00941). These findings suggest that EGCG, in addition to functioning as an antioxidant, may act as a potential regulator of flavonoid biosynthesis by modulating both signaling pathways and gene expression, thereby activating the plant’s intrinsic defense system. Polyphenolic compounds have been shown to upregulate genes in these pathways and promote the accumulation of flavonoid and phenolic compounds, thus improving plant tolerance to abiotic stresses such as heavy metals, ozone, and herbicides [41,42,43].

Validation by qRT-PCR confirmed that the expression trends of representative genes such as *PAL*, *F3H*, *CHI*, and *OMT* were highly consistent with transcriptome data, further supporting EGCG’s role in modulating the flavonoid biosynthesis pathway. Additionally, co-expression network analysis identified *PAL*, *C4H*, *APX*, and *GST* as hub genes with high connectivity, suggesting that EGCG may coordinate the regulation of antioxidant and secondary metabolic networks. This dual regulatory capacity offers promising potential for EGCG as a plant-protective agent and provides new insights into green approaches for pesticide residue mitigation.

## 4. Materials and Methods

### 4.1. Plant Materials and Treatments

Seeds of melon (*Cucumis melo* L. cv. ‘Tianbao’) were surface sterilized with 75% ethanol for 10 min and rinsed twice with deionized water. After germinating for 3 days, the seeds were sown into 56-cell nursery trays. Upon emergence of the first true leaf, uniform seedlings were transplanted individually into 3.8 L plastic pots filled with a peat-to-vermiculite mixture (3:1, *v*/*v*). Plants were grown in a controlled growth chamber under the following conditions: 600 μmol·m^−2^·s^−1^ photosynthetically active radiation (PAR), 14 h/10 h light/dark photoperiod, 27/19 °C (day/night) temperatures, and 70% relative humidity. When seedlings reached the four-leaf stage, EGCG pretreatment was applied by foliar spraying of 100 μM EGCG solution until runoff (droplet formation on the abaxial side of leaves). A control group was sprayed with an equal volume of deionized water containing 0.01% (*v*/*v*) organosilicon. Treatments were applied twice at 24 h intervals. 12 h after the second EGCG pretreatment, plants were subjected to one of four treatments: 1. Control treatment (CK): Sprayed with water containing 0.01% Tween^®^ 80 (Shanghai Aladdin Biochem Technology Co., Ltd., Shanghai, China). 2. EGCG treatment (EGCG): Sprayed with 100 μM EGCG (Sigma-Aldrich (Shanghai) Trading Co., Ltd., Shanghai, China) containing 0.01% Tween^®^ 80. 3. Glyphosate treatment (Gly): Sprayed with 3 g/L glyphosate (Beijing Green Agricultural Science and Technology Group Co., Ltd., Beijing, China). 4. Glyphosate and EGCG treatment (Gly-E): Sprayed with a mixture of 3 g/L glyphosate and 100 μM EGCG. All treatments were applied once at the four-leaf stage by foliar spraying on both sides of the leaves. Leaf samples were collected 48 h post-treatment. Each treatment included three biological replicates, with six plants per replicate.

### 4.2. Determination of Glyphosate Residues in Melon Leaves

Quantification of glyphosate residues in melon leaves was performed using liquid chromatography–tandem mass spectrometry (LC-MS/MS) equipped with a Triple Quad 5500 system (Shanghai AB Sciex Analytical Instrument Trading Co., Ltd., Shanghai, China). 1,2-13C, 15N-labeled glyphosate (purity ≥ 98.0%; Manhage Biotechnology Co., Ltd., Beijing, China) was used as an internal standard to ensure analytical accuracy. Fresh melon leaf tissue (10 g) was weighed into a 50 mL centrifuge tube and extracted with 50 mL of ultrapure water by vortexing at 400 rpm for 1 min, followed by ultrasonic extraction at 37 °C for 20 min. The mixture was centrifuged at 8000× *g* for 5 min at 25 °C, and the supernatant was transferred to a clean centrifuge tube. The residue was re-extracted with an additional 50 mL of ultrapure water under the same conditions. The two supernatants were combined and mixed thoroughly. A 10 mL aliquot of the combined extract was mixed with 10 mL of dichloromethane and vortexed for 2 min. After centrifugation at 12,000× *g* for 5 min at 25 °C, 2 mL of the aqueous phase was collected and passed through a glyphosate-specific solid-phase extraction (SPE) column, preconditioned sequentially with 2 mL of methanol and 2 mL of ultrapure water. The resulting eluate was collected for derivatization. For derivatization, 1 mL of eluate was mixed with 300 μL of FMOC-Cl in acetone (10 g·L^−1^) and 250 μL of 5% sodium borate solution, vortexed for 5 min, and then incubated at room temperature for 12 h. Prior to LC-MS/MS analysis, the sample was filtered through a 0.22 μm nylon membrane filter. Chromatographic separation was performed using a Thermo Scientific C18 column (2.1 mm × 50 mm, 1.8 μm) (Thermo Fisher Scientific Inc., Shanghai, China). The mobile phase consisted of 0.1% formic acid in water (A) and 0.1% formic acid in acetonitrile (B). The flow rate was maintained at 0.3 mL·min^−1^, with a column temperature of 40 °C and an injection volume of 15 μL.

### 4.3. Transcriptome Sequencing

Total RNA was extracted from melon leaves using the RNAprep Pure Plant Kit (Beijing Tiangen Biotech Co., Ltd., Beijing, China). RNA purity and integrity were assessed with a Nanodrop spectrophotometer (Thermo Fisher Scientific, Inc., Shanghai, China) and an Agilent 2100 Bioanalyzer (Agilent Technologies Inc., Santa Clara, CA, USA). Sequencing libraries were prepared using the NEBNext^®^ Ultra™ RNA Library Prep Kit (Bio-Rad Laboratories (Shanghai) Co., Ltd., Shanghai, China) and sequenced on an Illumina NovaSeq 6000 platform (Illumina, Inc., Foster City, CA, USA) with 150 bp paired-end reads. Raw sequencing data were quality-checked and trimmed using Trimmomatic (v 0.39) to remove adapter sequences and low-quality bases, yielding high-quality clean reads [44]. The clean reads were then aligned to the melon reference genome DHL92 (v 4.0) (http://cucurbitgenomics.org/v2/organism/23 (accessed on 30 May 2024)), available from the Cucurbit Genomics Database, using HISAT2 (v 2.2.1) [45]. Alignment results were further processed with Samtools (v 1.9) to retain only uniquely mapped reads with a mapping quality score greater than 10 [46]. The resulting consensus transcripts were merged and assembled into a non-redundant transcript set using StringTie (v 2.2.1). The assembled transcripts were compared and refined using gffcompare (v 0.12.6), and gene read counts were calculated using featurecounts [47]. Gene expression levels were normalized to fragments per kilobase of transcript per million mapped reads (FPKM).

### 4.4. Differential Gene Expression and Functional Enrichment Analysis

DEGs were identified using the DESeq2 package (v 1.48.2), a robust statistical tool for modeling count data from RNA-seq experiments [48]. Genes exhibiting a false discovery rate (FDR) less than 0.05 and an absolute log2 fold change (|log_2_FC|) ≥ 1 were considered significantly differentially expressed, indicating substantial and statistically supported shifts in expression between conditions.

To explore the biological significance of the DEGs, GO and KEGG enrichment analyses were performed using the clusterProfiler package (v 4.16.0) [49]. This analysis systematically identified overrepresented biological processes, molecular functions, cellular components (GO), and metabolic or signaling pathways (KEGG) among the DEGs.

Statistical enrichment was determined using adjusted *p*-values to control multiple testing, ensuring reliable identification of significantly enriched categories. This approach facilitates a deeper understanding of the underlying biological mechanisms and pathways potentially involved in the condition or treatment under investigation.

### 4.5. Quantitative Real-Time PCR (qRT-PCR) Validation

To investigate the expression profiles of antioxidant-related genes, total RNA was extracted from melon leaves using the RNAperp Pure Plant Plus Kit (Beijing Tiangen Biotech Co., Ltd., Beijing, China) according to the manufacturer’s instructions. First-strand cDNA synthesis was performed with the Evo M-MLV RT Kit with gDNA Clean (Accurate Biotechnology, Changsha, China), using 1 ng of total RNA per reaction. All gene-specific primers used in this study were derived from our previous research [18,28]. qRT-PCR was performed on a LightCycler^®^ 480 II System (Thermo Fisher Scientific Inc., Shanghai, China). The thermal cycling protocol was as follows: 95 °C for 3 min (initial denaturation), followed by 40 cycles at 95 °C for 10 s (denaturation), 58 °C for 45 s (annealing), and 72 °C for 30 s (extension). The melon elongation factor 1-alpha gene (EF1α, *MELO3C003128.2*) was used as the internal control. Relative gene expression was calculated using the 2^–ΔΔCt^ method as described by Livak and Schmittgen [50].

### 4.6. Statistical Analysis

Data were statistically evaluated using one-way analysis of variance (ANOVA) with SPSS software version 22.0 (International Business Machines Corporation, Chicago, IL, USA). When significant differences were observed, Duncan’s multiple range test was applied for post hoc comparisons. In addition, least significant difference (LSD) tests were used to assess pairwise comparisons among group means, with statistical significance set at *p* ≤ 0.05. All graphical illustrations were created using OriginPro version 8.6 (OriginLab Corporation, Northampton, MA, USA).

## 5. Conclusions

This study provides clear evidence that exogenous EGCG application enhances the degradation of glyphosate in melon and reduces its tissue accumulation, potentially through selective regulation of antioxidant and secondary metabolic gene networks. Transcriptomic analyses revealed that EGCG mitigates glyphosate-induced stress by attenuating excessive detoxification gene activation while enhancing flavonoid biosynthesis and hormone-responsive pathways. The dual role of EGCG—as a modulator of oxidative defense and a promoter of metabolic adaptation—underscores its potential as a green and sustainable approach to reduce herbicide residues in crops. These results offer new insights into the utilization of natural polyphenols for integrated pesticide residue management in horticulture.

## Figures and Tables

**Table 1:** Summary of RNA-seq data quality and filtering statistics for melon leaf samples under different treatments.

Sample	RawReads (M)	RawBases (G)	CleanReads (M)	CleanBases (G)	ValidBases (%)	Q30 (%)	GC (%)
CK-1	48.34	7.25	48.26	7.09	97.84	93.14	45.98
CK-2	50.65	7.6	50.56	7.44	97.94	94.91	45.59
CK-3	47.09	7.06	47.03	6.93	98.12	94.91	45.7
EGCG-1	48.92	7.34	48.84	7.19	98.03	95.18	45.96
EGCG-2	50.19	7.53	50.11	7.35	97.63	94.6	45.96
EGCG-3	49.9	7.48	49.82	7.34	98.11	95.07	45.93
Gly-1	50.39	7.56	50.3	7.35	97.22	94.97	45.34
Gly-2	50.21	7.53	50.1	7.34	97.41	94.6	45.25
Gly-3	47.58	7.14	47.5	6.97	97.7	94.74	45.14
Gly-E-1	48.87	7.33	48.77	7.14	97.46	94.67	45.38
Gly-E-2	48.71	7.31	48.61	7.13	97.58	95.18	45.46
Gly-E-3	47.47	7.12	47.39	6.99	98.15	95.36	45.51

## Data Availability

All data are available on request to the corresponding author.
